# Chaotic dynamics as a possible mechanism of rapid change of hippocampal local field activity between theta rhythm and large irregular activity

**DOI:** 10.1186/1471-2202-13-S1-P189

**Published:** 2012-07-16

**Authors:** Keita Tokuda, Yuichi Katori, Kazuyuki Aihara

**Affiliations:** 1 Department of Mathematical Informatics, Graduate School of Information Science and Technology, The University of Tokyo, 7-3-1 Hongo, Bunkyo-ku, Tokyo, Japan; 2 FIRST, Aihara Innovative Mathematical Modelling Project, JST; 3 Institute of Industrial Science, The University of Tokyo, 4-6-1 Komaba, Meguro-ku, Tokyo 153-8505, Japan

## 

Here we propose a possible role of chaotic dynamics in the generation of two distinctive rhythm patterns of local field potential of the hippocampus; namely the theta rhythm and large irregular activity (LIA). The basic idea is that the rapid alternation of the state between theta rhythm and LIA can be described as bifurcation of the attractor between limit cycle and chaos.

**Figure 1:**
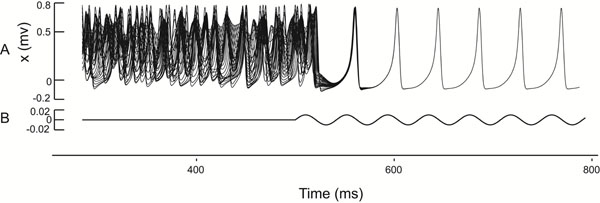
Time evolution of the variables of the model representing the membrane potentials of the neurons **(A)** and the amplitude of the external current representing the septal inputs to the hippocampus **(B)**.

It is well known that the hippocampus has two distinctive states described by the characteristic activity of the local field potential, the theta rhythm and LIA. The theta rhythm is a highly periodic activity thought to play an important role in learning and retrieving process, whereas LIA is a irregular field activity with sharp ripple complex, which is thought to play an important role in the consolidation process of old memories. However, the underlying mechanism of realizing these two distinctive field oscillations embedded in the same network remains unknown. On the other hand, Katori et al. reported that transitions between synchronous and asynchronous oscillatory state can be realized with gap junction-coupled simple conductance-based model neurons [1]. Further, Tsuda et al. reported that model network composed of simple class 1 model neurons connected with gap junctions show both asynchronous chaotic behavior and synchronous behavior with fixed model parameters [2]. Here we model the network of hippocampal interneurons with this model, taking into consideration that the hippocampal interneurons are thought to have gap junctions [3]. We compare the dynamics of the model with in vivo electrophysiological data and show that this model reproduces qualitative characteristics of the experimental data. Moreover, we show that oscillatory ascending activity mimicking the medial septal projection, which is known to project to the hippocampal interneurons and is widely assumed to be the theta rhythm pacemaker, can entrain the model network and change the network state rapidly to synchronized state (see Figure 1). We conduct bifurcation analysis and show how all-synchronized saddle periodic orbit is stabilized to stable periodic orbit by external input.
